# Serosurvey of Toxoplasmosis Among Drug Users in Fars Province, Southern Iran: A Cross‐Sectional Study

**DOI:** 10.1002/hsr2.73057

**Published:** 2026-08-26

**Authors:** Mohammad Reza Cheraghi, Mina Zarrabi, Sara Shahriarirad, Sahel Rohampour, Ghazal Shayan, Mostafa Omidian, Elham Rezaee, Jamal Sarvari, Abolfazl Gheshlaghi, Qasem Asgari, Bahador Sarkari

**Affiliations:** ^1^ Student Research Committee Shiraz University of Medical Sciences Shiraz Iran; ^2^ Department of Parasitology and Mycology, School of Medicine Shiraz University of Medical Sciences Shiraz Iran; ^3^ Department of Microbiology, School of Medicine Shiraz University of Medical Sciences Shiraz Iran; ^4^ Basic Sciences in Infectious Diseases Research Center Shiraz University of Medical Sciences Shiraz Iran

**Keywords:** Iran, seroprevalence, substance use disorder, *Toxoplasma*

## Abstract

**Background and Aims:**

*Toxoplasma gondii* is a widespread zoonotic protozoan that poses significant health risks, particularly to immunocompromised individuals. Although generally asymptomatic, toxoplasmosis can lead to severe neurological complications and behavioral disorders. This infection can be transmitted through various routes, including sharing needles and/or syringes with someone who is infected. The current cross‐sectional study aimed to determine the prevalence of toxoplasmosis among drug users in Fars Province in the south of Iran.

**Methods:**

Subjects of the study were 1400 drug users. Blood samples were collected from each individual from June 2023 to April 2024. *T. gondii* antigens were prepared from the parasite's tachyzoites and used in an ELISA system to detect anti‐*Toxoplasma* antibodies. The data were analyzed to explore the possible association between *Toxoplasma* seropositivity and participants' demographics.

**Results:**

The majority (52.7%) of the participants were aged 40–60 years, with 83.4% living in urban areas. Males constituted 92.8% and females 7.2% of the subjects. Anti‐*Toxoplasma* antibodies were detected in the sera of 189 individuals, corresponding to a seroprevalence of 13.5% (95% CI, 9.5%–15.4%). Notably, 89.9% of those using injection methods for substance use were infected. Moreover, 94.2% of the infected individuals were male. Our findings revealed no association between *Toxoplasma* seropositivity and the demographic or method of drug use in patients with substance use disorder in Fars Province in the south of Iran.

**Conclusion:**

Findings of the current study underscore the need for targeted public health interventions to address the risk of toxoplasmosis in this vulnerable population, particularly among those who use injection drugs. Further research is warranted to explore the underlying factors contributing to *Toxoplasma* infection in this population and to develop effective prevention strategies.

## 1 Introduction

*Toxoplasma gondii* is a globally widespread protozoan parasite that can infect both animals and humans [1]. It can infect and reside in a wide range of warm‐blooded animals, such as mammals and birds, but is primarily spread in the environment by its only known definitive host, the Felidae family [1, 2]. Toxoplasmosis is a food‐borne infection transmitted primarily through the ingestion of sporulated oocyst‐contaminated water, food, or tissue cysts from undercooked meat [3]. It can also be transmitted through parenteral transmission, transplacental transmission, or organ transplant [4]. Toxoplasmosis is typically asymptomatic but can lead to severe consequences, such as neurological complications and even death, particularly for fetuses and immunocompromised individuals [5, 6]. There have also been reports of behavioral changes and neuropsychological disorders, including bipolar disorder and schizophrenia, following infection with *Toxoplasma* [7]. There has been an increasing interest in exploring the association between these disorders and toxoplasmosis. Although the exact cause is not completely understood, these disorders are partially attributed to changes in dopamine signaling pathways [8].

Over one‐third of the world population is infected with toxoplasmosis, although its prevalence can vary from place to place depending on general hygiene, socioeconomic status, climate, and geographical location [9, 10]. According to a systematic review, Africa had the highest prevalence, while Asia had the lowest average prevalence rate of 16.4% among continents [10]. In a systematic review by Daryani et al., the seroprevalence of toxoplasmosis in Iran has been estimated at 39.3%. However, the prevalence rate in different regions varies greatly; for instance, the prevalence rate in the southern region of Iran is between 26% and 46.9%, while in the northern part of the country, it can range from 14% to as high as 66% [11]. The seroprevalence of toxoplasmosis in high‐risk populations in Iran, such as pregnant women and immunocompromised individuals, has been reported as 41% and 45.1%, respectively [12, 13]. In another study in Iran on women eligible for pregnancy, the infection rate was reported as 39.9% [14]. The seroprevalence in the blood donor population as well as between hemodialysis patients in Iran has been evaluated and estimated at 19.3% and 58%, respectively [15, 16].

Drug addiction is a serious problem in many countries, especially in Iran. Depending on the type of drug used, it can have various effects, such as decreased immunity [17]. These drugs are associated with increased dopaminergic activity in the brain, and *Toxoplasma* can also affect the brain's reward pathway neurotransmitters and dopaminergic signaling, indirectly or directly, thus potentially being a risk factor for substance use in individuals [7, 18, 19]. In a study in the US, anti‐*Toxoplasma* antibodies in patients with substance use disorder (SUD) were about 20% [18]. The infection rate in high‐risk pregnant women with SUD and individuals with opioid addiction was recently evaluated in Iran and reported as 37.5% and 34.3%, respectively [17, 20]. In a different study on smokers in Shiraz, Fars Province, 20.7% of the smokers were positive for *T. gondii* infection [7].

Drug users often engage in behaviors that increase their risk of exposure to *T. gondii*. These behaviors may include poor hygiene practices, consumption of undercooked or contaminated food, and close contact with environments contaminated with oocysts from cat feces. Moreover, many drug users may have compromised immune systems due to the direct effects of drug use or co‐infections, such as HIV. This immunosuppression can make them more susceptible to severe manifestations of toxoplasmosis.

Understanding the seroprevalence of toxoplasmosis in drug users can help in assessing the potential for transmission within this population and to the broader community. Identifying the prevalence and risk factors associated with toxoplasmosis in drug users can inform targeted public health interventions, including education, screening, and treatment programs. There is limited data on the seroprevalence of toxoplasmosis among drug users in Iran, including Fars Province in the south of the country. Considering these points, the current study was conducted to determine the seroprevalence of toxoplasmosis among drug users in Fars Province, southern Iran.

## 2 Methods

### 2.1 Study Area and Subject Recruitment

This cross‐sectional study was conducted from June 2023 to April 2024 and included 1400 drug users from Fars Province in southern Iran (coordinates 29°6′15.7727″ N, 53°2′45.2148″ E). Approximately 5000 individuals in the region were identified as drug users during the study period. Of these, 1400 participants were recruited based on predefined inclusion and exclusion criteria. Individuals were excluded if they were not classified as drug users, were unwilling or unable to provide informed consent or a blood sample, submitted compromised samples (e.g., due to improper handling or hemolysis), or were not residents of Fars Province. Participants were recruited through convenience sampling. This nonprobability sampling approach was employed due to logistical considerations and the nature of the target population.

### 2.2 Blood Sampling

All participants had existing records at the health center or the AIDS control center. Blood samples were collected during their routine visits for required clinical evaluations or treatment follow‑up. Aseptic blood samples (5 mL) were collected from each subject and transported to the Parasitology and Mycology Department at Shiraz University of Medical Sciences (SUMS), Shiraz, Iran, in a cold box. The sera were then separated using a centrifuge and stored at −20°C until use. Sociodemographic information, including sex, age, area of residence, type of drug use, and so forth, was collected while sampling.

### 2.3 Preparation of *T. gondii* Antigen

The RH strain of *T. gondii* available in the Parasitology and Mycology Department of the Shiraz School of Medicine was utilized for antigen preparation. Approximately 6 × 10^6^ tachyzoites in 0.5 mL of phosphate‐buffered saline were intraperitoneally injected into a BALB/c mouse. After 72 h, the proliferated parasites were aspirated from the mouse's peritoneum using a syringe. The aspirated parasites were centrifuged at 200 g for 4 min to sediment leukocytes and cellular debris. The supernatant containing the *Toxoplasma* parasites was then separated and centrifuged again at 800 g to sediment the tachyzoites. Following sedimentation, the parasites were washed twice with PBS.

To dissociate the parasites, 0.1 g/mL trypsin was added, and the solution was gently mixed until the cells were completely separated. The number of parasites was counted using a Neubauer chamber, and the solution was adjusted to a concentration of 50 million *T. gondii* parasites per milliliter. To lyse the parasites, two cycles of freeze‐thaw and sonication with ultrasonic waves were employed. The sonication was performed at 50 watts, 5 times for 30 s with 20‐s intervals. The tachyzoites were examined under a microscope to ensure complete lysis. The antigen solution was then centrifuged at 1300 g at 4°C for 30 min. The supernatant containing the parasite antigens was separated, and after measuring the protein concentration, it was stored at −20°C until use.

### 2.4 ELISA for the Detection of Anti‐*Toxoplasma* Antibodies

An in‑house ELISA for the detection of anti‑*Toxoplasma* antibodies was conducted according to established protocols described in our earlier study [21]. Initially, the optimal concentrations of antigen, serum, and secondary antibody were determined using the ELISA checkerboard method. For the ELISA procedure, the 96‐well ELISA microplate (SPL Life Sciences) was coated with the *Toxoplasma* antigen in a coating buffer (0.1 M carbonate/bicarbonate buffer, pH 9.6) at a concentration of 5 µg/mL and incubated overnight at 4°C. After incubation, the antigen solution was removed, and the wells were washed five times with PBST (PBS with 0.05% Tween 20) using an ELISA washer. Next, 100 µL of blocking buffer (3% nonfat skimmed milk in PBST) was added to each well and incubated at room temperature for 2 h. Following another wash, 100 µL of serum samples diluted 1:100 was added to each well, and the plate was incubated for 1 h. The wells were washed again and 100 µL of peroxidase‐conjugated antibody (Sigma‐Aldrich: goat antihuman IgG antibody), diluted 1:3000 in PBST, were added to each well. The plate was incubated at room temperature for 1 h. After this incubation, the wells were washed five times as before. Finally, 100 µL of OPD substrate solution containing 3% hydrogen peroxide was added to each well, and the plate was incubated at room temperature for 15 min. The optical density (OD) was then read using an ELISA reader at wavelengths of 450 and 630 nanometers. The cut‑off value was determined by calculating the mean OD of confirmed negative samples plus or minus two standard deviations. The negative samples used for this calculation were those whose negativity had been verified using a commercial diagnostic kit.

All seropositive cases were reevaluated for anti‐*Toxoplasma* antibodies using a commercial *Toxoplasma* IgG ELISA kit (Pishtazteb Diagnostics, Iran), according to the manufacturer's instructions. The commercial assay served as a benchmark for standardization and reproducibility.

### 2.5 Statistical Analysis

Analyses of the data were performed in accordance with the guidelines recommended by Assel et al. (2019) [22]. The demographic data of the study participants and the test outcomes were input into SPSS (version 26) software. The chi‐square test was employed to examine the association between *Toxoplasma* seropositivity and the participants' demographic characteristics. Multivariate logistic regression analysis was used to find out the independent risk factors for *Toxoplasma* seropositivity in the recruited subjects.

### 2.6 Ethical Consideration

The study was approved by the Ethical Committee of Shiraz University of Medical Sciences (Ref. No. IR.SUMS.MED.REC.1402.308), and informed consent was obtained from each participant. The Ethics Committee also reviewed and approved the use of mice for parasite maintenance and antigen preparation.

## 3 Results

A total of 1400 SUD patients were recruited in this study. The male population constituted 92.8% of participants (*n* = 1299), while 7.2% (*n* = 101) were female. The mean age of the participants was 44.59 ± 12.50 years (range 17–73). Most participants were between 51 and 60 years of age (28.3%, *n* = 396) and resided in urban areas (83.4%). Out of the 1400 subjects, 189 (13.5%; 95% CI, 9.5%–15.4%) tested positive for anti‐*Toxoplasma* antibodies.

There was no statistically significant association between age groups or location of residence and seropositivity to toxoplasmosis (*p* > 0.05). Among all participants, 89.9% of those who used injection as their method of drug use were seropositive for *Toxoplasma* infection. However, there was no significant association between the method of drug use and toxoplasmosis seropositivity (*p* = 0.15). Additionally, 13.7% of males (*n* = 178) and 10.9% of females (*n* = 11) tested positive for anti‐*Toxoplasma* antibodies, but there was no significant association between *Toxoplasma* seropositivity and gender (*p* = 0.43). Multivariate logistic regression analysis indicated that only the gender with OR = 2.03 (95% CI, 1.33–3.72; *p* = 0.032) was associated with seropositivity to *Toxoplasma*. The demographic features of the studied subjects and their seropositivity to toxoplasmosis are presented in Table 1.

**Table 1:** Sociodemographic and drug use method and relative *Toxoplasma* seropositivity in Fars Province, Southern Iran.

Variables	Frequency (%)	Positive for anti‐*Toxoplasma*	*p* value
		Antibodies (%)	
*Gender*			0.426
Male	1299 (92.8)	178 (94.2)	
Female	101 (7.2)	11 (5.8)	
*Age*			0.127
≤ 30	219 (15.6)	38 (20.1)	
31–39	345 (24.6)	45 (23.8)	
41–60	312 (22.3)	36 (19.0)	
51–60	396 (28.3)	59 (14.9)	
≥ 61	128 (9.1)	11 (8.6)	
*Place of residence*			0.575
Urban	1167 (83.4)	157 (16.9)	
Rural	233 (16.6)	32 (83.1)	
*Drug use method*			0.149
Injection	179 (12.8)	18 (9.5)	
Other means	1221 (87.2)	171 (90.5)	

## 4 Discussion

*T. gondii* is an underestimated threat that can cause an array of physical, psychological, and ocular disorders. In this study, the seroprevalence of *T. gondii* in the population of patients who use drugs in the south of Iran was assessed, revealing a seropositivity rate of 13.5%. Compared with another study on SUD patients in Iran, our seropositive population is reported to be lower than their results (34.5%) [17].

Drugs are known to increase dopaminergic activity in the brain, and *Toxoplasma* can similarly influence the brain's reward pathway neurotransmitters and dopaminergic signaling, both directly and indirectly [7, 18, 19]. Increased dopamine levels in the brain have been reported to have an inverse effect on substance use, decreasing one's tendency towards drugs [7, 18]. There are not many studies on the effects of different types of drugs on toxoplasmosis. In a survey by Sharifzadeh et al., comparing patients with SUD with other individuals, it was concluded that injecting drug users have a higher tendency to develop infection due to regular contact with contaminated syringes. However, the authors noted that their sample of injecting drug users was relatively small [17]. Another study also suggested that injection of narcotic drugs is an important criterion related to higher toxoplasmosis infection [23]. Comparatively, our findings showed a lower prevalence rate in drug users who inject drugs. Nonetheless, participants' inclination to provide socially acceptable responses might have affected the results. Additionally, the lack of specificity regarding drug use methods other than injection in the present analysis warrants a more detailed study on this matter.

Across several studies conducted in Fars Province, the prevalence of *T. gondii* infection shows substantial variation depending on the population examined, ranging from 3.8% to 45.8% [15, 24, 25, 26]. In the current study, we found a toxoplasmosis seroprevalence of 13.5% among drug users in Fars Province, a rate that falls within the lower–middle range compared with previous reports from the region. This prevalence is lower than that observed in breast cancer patients (45.8%) [25] and hemodialysis patients (18%–25%), and slightly below the rate reported among blood donors (19.3%) [15], but higher than the very low prevalence documented in rural children (3.8%) [24]. These differences likely reflect variation in age structure, underlying health conditions, lifestyle factors, and environmental exposure across populations. Clinical groups such as breast cancer and hemodialysis patients tend to be older and may have greater cumulative exposure or immune alterations that increase susceptibility. Blood donors, typically healthy adults, show moderate prevalence consistent with general community exposure. In contrast, rural children have limited lifetime exposure, explaining their low rates. The 13.5% prevalence among drug users may be influenced by socioeconomic factors, unstable living conditions, or reduced hygiene practices, but may also be moderated by limited contact with typical transmission routes, such as raw meat consumption or soil exposure.

Although our results indicated a higher seroprevalence of toxoplasmosis among urban residents, no meaningful correlation was found between participants' residential location and seropositivity to toxoplasmosis. In contrast, another study on the general seroprevalence of toxoplasmosis among Iranians documented a higher seroprevalence for toxoplasmosis amongst rural participants [11]. Additionally, the lack of correlation between participants' location and toxoplasmosis prevalence may be due to the migration of rural citizens to urban areas in search of more favorable life conditions. As IgG antibody titers tend to remain the same in a previously exposed person to the parasite during their lifetime, this can result in interpretations being more complex [11]. Furthermore, a number of organizations taking care of our participants with SUD were located in urban areas, thus resulting in some individuals from the rural regions being reported as urban participants. It is recommended to conduct a study with fewer location‐related limitations for more accurate results.

Another risk factor for the prevalence of *Toxoplasma* infection is age. Our findings demonstrated no meaningful association between toxoplasmosis seroprevalence and age. In a study among patients with SUD and nonaddicted individuals, age has been suggested to have a significant effect on toxoplasmosis seropositivity in the population with SUD [17]. Other studies have also indicated that age could be a potentially influential risk factor regarding the *Toxoplasma* infection [7, 11, 17, 20, 27, 28, 29]. The reason is generally suggested to be longer exposure to the parasite as one becomes older [27, 29].

Gender can be a risk factor in various diseases, although in this particular infection, our observations align with other studies that show no correlation between sex and *Toxoplasma* seropositivity [29, 30, 31]. This might be attributed to the fact that there are enough factors regarding lifestyle, activities, and general occupations provided for both genders to become infected with *Toxoplasma*. Our study's outcome regarding gender is consistent with the literature although since more than half of our samples consisted of male population further research can be beneficial. Our study has several limitations. First, most samples were reported to be from urban areas, which may not accurately represent the seroprevalence of *T. gondii* in rural populations. Second, the study predominantly consisted of male participants, leading to a lack of gender diversity. Third, important factors such as living conditions, pet ownership, and occupation were not considered in this study. These variables could significantly influence the risk of *T. gondii* infection, and their exclusion limits the depth of the analysis. Social norms and rules might have influenced participants to withhold the complete truth, potentially leading to underreporting or misreporting of behaviors and conditions relevant to *T. gondii* exposure. Furthermore, the reliance on a home‑made ELISA for diagnosis and the absence of an assessment of infection‑related risk factors constitute further limitations of the present study.

## 5 Conclusion

This cross‐sectional study aimed to determine the prevalence of toxoplasmosis among drug users in Fars Province, southern Iran. The seroprevalence of toxoplasmosis was found to be 13.5% among the 1400 participants. The majority of the subjects were male and resided in urban areas. Although the prevalence of *Toxoplasma* infection was higher in males than in females, gender was not significantly associated with seropositivity. Also, the study did not find a definitive link between the infection and demographic or behavioral factors. Interestingly, the prevalence of toxoplasmosis in patients with SUD is considerably lower than in the general population. Further research is warranted to explore the underlying factors contributing to *Toxoplasma* infection in this population and to develop effective prevention strategies. Collecting more detailed data, such as the specific types of drugs used, could improve the effectiveness of future public health strategies and assist health policymakers.

## Author Contributions

**Mohammad Reza Cheraghi:** data curation, investigation. **Mina Zarrabi:** data curation, investigation. **Sara Shahriarirad:** data curation, investigation, writing – original draft. **Sahel Rohampour:** data curation, investigation. **Ghazal Shayan:** data curation, investigation. **Mostafa Omidian:** data curation, investigation. **Elham Rezaee:** methodology, writing – review and editing, data curation. **Jamal Sarvari:** conceptualization, methodology, formal analysis, writing – review and editing. **Abolfazl Gheshlaghi:** data curation, investigation. **Qasem Asgari:** conceptualization, methodology, formal analysis. **Bahador Sarkari:** conceptualization, methodology, formal analysis, writing – review and editing.

## Conflicts of Interest

The authors declare no conflicts of interest.

## Transparency Statement

The lead author Bahador Sarkari affirms that this manuscript is an honest, accurate, and transparent account of the study being reported; that no important aspects of the study have been omitted; and that any discrepancies from the study as planned (and, if relevant, registered) have been explained.

## Data Availability

The data that support the findings of this study are available from the corresponding author upon reasonable request.
